# Electrochemical Catheter Hub Operated by a Wearable Micropotentiostat Prevents *Acinetobacter baumannii* Infection In Vitro

**DOI:** 10.1002/bit.28990

**Published:** 2025-04-15

**Authors:** Majid Al‐Qurahi, Derek Fleming, Won‐Jun Kim, Ibrahim Bozyel, Robin Patel, Haluk Beyenal

**Affiliations:** ^1^ The Gene and Linda Voiland School of Chemical Engineering and Bioengineering Washington State University Pullman Washington USA; ^2^ Division of Clinical Microbiology, Department of Laboratory Medicine and Pathology Mayo Clinic Rochester Minnesota USA; ^3^ Department of Electrical and Computer Engineering Worcester Polytechnic Institute Worcester Massachusetts USA; ^4^ Division of Public Health, Infectious Diseases, and Occupational Medicine, Department of Medicine Mayo Clinic Rochester Minnesota USA

**Keywords:** central line‐associated bloodstream infection, central venous catheter, electrochemical catheter hub, hypochlorous acid, infection prevention, wearable micropotentiostat

## Abstract

Intraluminal infection of central venous catheters, used for long‐term treatment, can result in central line‐associated bloodstream infection (CLABSI). These infections can be challenging to prevent and treat due to formation of biofilms within catheter lumens, which shield bacteria from the human immune response and conventional antimicrobial therapies. Preventing bacterial colonization of catheter hubs is a strategy to prevent CLABSI. To address this, we developed a nonantibiotic, animal‐ready electrochemical catheter hub (e‐catheter hub), operated by a wearable, battery‐powered micropotentiostat (MP), that internally generates tunable hypochlorous acid (HOCl) for preventing intraluminal infection. The design evaluated three different electrode materials—titanium, platinum, and gold—for HOCl generation and biocidal activity, using working and counter electrodes of the same materials and a silver/silver chloride‐plated wire as a quasi‐reference electrode. e‐catheter hubs operated by MPs at 1.5 V_Ag/AgCl_ for 3 h generated HOCl, reducing *Acinetobacter baumannii* ATCC‐17978 below the detection limit (average reduction of 4.40 ± 0.05 log_10_ CFU/mL). The efficacy of e‐catheter hubs operated by MPs in generating HOCl and achieving biocidal activity is comparable to that of a commercial potentiostat. This study represents the first step in developing a localized, nonantibiotic strategy to mitigate CLABSI risk.

## 1 Introduction

Bloodstream infections (BSIs) are significant and growing global health challenges, contributing to morbidity and mortality (Costa and Carvalho 2022). In the United States, BSIs account for an estimated 536,000 to 628,000 cases and 72,000 to 85,000 deaths annually, and rank among the top seven leading causes of death in various North American and European countries (Goto and Al‐Hasan 2013). Central venous catheters (CVCs) are implicated in ~20% of BSIs, causing a condition called central line‐associated bloodstream infection (CLABSI) (Wassil et al. 2007). CVCs are used for medical treatment, particularly in critically ill patients who require fluid and/or medication administration or monitoring in intensive care units (ICUs). Beyond critical care, CVCs are used in non‐critically ill patients requiring long‐term therapies, such as antibiotic administration, hemodialysis, or parenteral nutrition (Alshahrani et al. 2023).

CLABSIs are major contributors to healthcare‐associated infections, leading to an estimated 28,000 additional deaths annually and imposing healthcare costs in the United States exceeding $2 billion per year (Sagana and Hyzy 2013). The COVID‐19 pandemic led to a rise in CLABSIs due to prolonged CVC use, ICU admissions, and critical care procedures. These factors contributed to higher mortality rates, longer hospital stays, and increasing healthcare costs compared to pre‐pandemic levels or non‐COVID‐19 ICU patients (Fakih et al. 2022; LeRose et al. 2021; Louis et al. 2021; Massart et al. 2021).

CLABSIs arise through two primary routes: extraluminal and intraluminal colonization (Frasca et al. 2010). Extraluminal infections occur primarily in short‐term CVCs, when microorganisms migrate from catheter insertion sites along the external catheter surface towards the bloodstream. Intraluminal infections occur in the catheter hub and lumen, often due to microbial contamination from improper handling of the catheter hub during connections or disconnections, which introduce microorganisms into the lumen (Frasca et al. 2010). Intraluminal infections are particularly risky with CVCs in place for more than 30 days, for which colonization rates can reach up to 40% (Mermel 2011). Microbial colonization within the lumen promotes biofilm formation – microbial communities embedded in a self‐produced extracellular matrix that adheres to the catheter surface (Gominet et al. 2017). The complex structure of biofilms hinders the activity of antimicrobial agents and immune cells, rendering biofilm‐associated infections challenging to treat using conventional strategies (Grooters et al. 2024; Shree et al. 2023).

Biofilm formation can begin as early as the day of catheter placement, with location and extent influenced by the catheter's surface composition (Gominet et al. 2017). For CVCs in place for fewer than 10 days, biofilms typically form on the external surface (Donlan 2001). In contrast, for long‐term CVCs (in place > 30 days), biofilms are more commonly found in the lumen (Mirghani et al. 2022). The pathogens most associated with these biofilm‐associated infections have been defined through surveillance studies. Between 2011 and 2014, coagulase‐negative staphylococci (CoNS) and *Staphylococcus aureus* (both frequently methicillin‐resistant), *Enterococcus* species, *Candida* species, and Gram‐negative bacteria such as *Escherichia coli*, *Klebsiella pneumoniae*, and *Pseudomonas aeruginosa*, were reported as the most common pathogens responsible for CLABSIs in the United States (Mermel et al. 2009; Weiner et al. 2016). These microorganisms are well‐adapted to biofilm formation, which contributes to their persistence and resistance to treatment.

While current approaches for CLABSI prevention focus on aseptic techniques to reduce extraluminal and intraluminal infections, intraluminal infections remain prevalent. Strategies, such as antimicrobial impregnated catheters, have been developed to address intraluminal infections. Available antimicrobial impregnated catheters contain either chlorhexidine/silver sulfadiazine (CHSS) or minocycline/rifampin (MR) (Wassil et al. 2007). CVCs impregnated with MR are associated with lower infection rates compared to those with CHSS due to available MR catheters being coated on external and internal surfaces (Wassil et al. 2007). MR coated catheters have proven to be most effective for patients needing prolonged catheterization; however, they come at a cost (Marciante et al. 2003). Another approach is to use antimicrobial catheter locks, with antibiotic or antiseptic (e.g., alcohol, taurolidine) agents (Zhang et al. 2019), or citrate‐based catheter lock solutions (Shah et al. 2002), which have demonstrated promising results in limiting intraluminal bacterial colonization. However, catheter lock solutions can theoretically be associated with toxic effects associated with long‐term exposure. In addition, catheter lock solutions with antibiotics can promote the selection for antibiotic‐resistance.

In summary, despite advancements in CLABSI prevention, current strategies remain limited, particularly for addressing intraluminal infection and biofilm‐associated resistance. Traditional approaches face several barriers: (1) increasing antibiotic resistance, which limits the activity of conventional antimicrobial therapies; (2) reliance on manual interventions, which can introduce pathogens during catheter manipulation; and (3) absence of a dynamically controlled and sustained antimicrobial delivery mechanism within catheter lumens, which can deliver varying drug levels over time, impact the efficacy of antimicrobial prevention strategies.

Hypochlorous acid (HOCl) is a promising alternative to traditional antibiotics due to its potent antimicrobial properties (Maher M 2023). HOCl, a fast‐acting antimicrobial agent produced by the immune system (Andrés et al. 2022), which targets a range of microorganisms, including bacteria, fungi, and viruses, by interacting with sulfur‐containing amino acids, lipids, nucleic acids, and membrane components, leading to cellular damage (da Cruz Nizer et al. 2020). HOCl disrupts biofilms by oxidizing extracellular polymeric substances (EPS), damaging bacterial membranes, and impairing cellular functions. However, subinhibitory concentrations can stimulate biofilm formation by inducing release of intracellular material, particularly extracellular DNA (eDNA), a key component of the EPS matrix. Additionally, exposure to sub‐lethal concentrations can trigger adaptive bacterial responses, allowing cells to recover from oxidative damage and form more robust biofilms (da Cruz Nizer et al. 2020). This resilience underscores the importance of using HOCl at bactericidal concentrations to prevent unintended selection of stronger, more persistent biofilm‐forming bacterial populations. Consistent with these principles, our prior work with electrochemical e‐scaffolds (e‐scaffolds) (Raval et al. 2021) demonstrated that 27 bacterial isolates exhibited mean HOCl minimum inhibitory concentration (MIC) values of 0.5–1.99 mM, below concentrations toxic to mammalian cells (~15.12 mM). Notably, under these conditions, repeated exposure to HOCl‐producing e‐scaffolds did not lead to the development of resistance. The current direct therapeutic application of HOCl is limited due to rapid degradation and challenges with storage and handling. HOCl solutions are unstable when exposed to ultraviolet light, air, and elevated temperatures (≥ 25°C). Additionally, maintaining stable concentrations of HOCl requires storage in the dark and under cool conditions (< 10°C) (Ishihara et al. 2017). These factors complicate its use as a direct solution, necessitating the development of stabilization methods or alternative delivery mechanisms.

Electrochemical technologies offer a promising approach for In Situ generation of HOCl. The ability to generate HOCl electrochemically and efficacy in preventing infections and biofilm formation have been reported. For example, our group generated HOCl electrochemically through e‐scaffolds (Flurin et al. 2021; Kiamco et al. 2019) and electrochemical bandages (e‐bandages) (Fleming et al. 2024; Mohamed et al. 2023). These technologies utilize electrochemical principles to generate HOCl locally and have been shown to reduce bacterial biofilms In Vitro and in murine wounds. Briefly, HOCl is generated electrochemically through electrolysis of a chloride‐containing solution, such as NaCl or buffered physiological saline (BPS) (Cano et al. 2021; Kiamco et al. 2019; Mohamed et al. 2023). When NaCl dissolves in water, it dissociates into sodium ions (Na^+^) and chloride ions (Cl^−^). In an electrolytic cell, Cl^−^ ions are oxidized at the surface of the working electrode (Trasatti 1987) when the applied formal potential exceeds 1.33 V_Ag/AgCl_ (pH = 5.5), forming chlorine gas (Cl_2_), as shown in Equation 1 (Haynes 2014). Chlorine gas rapidly dissolves in water, forming a mixture of HOCl and hypochlorite (Equations 2 and 3).

(1) $$2Cl^{-}\to Cl_{2}+2e^{-}\;E^{o^{\prime }}=1.33\;V_{Ag/AgCl}\;$$

(2) $$Cl_{2}+H_{2}O↔\mathrm{HOCl}+Cl^{-}+H^{+}$$

(3) $$\mathrm{HOCl}↔\mathrm{OC}l^{-}+H^{+}$$

The success of the previously mentioned technologies has enabled their adaptation for biomedical applications aimed at addressing CLABSI prevention by targeting intraluminal infection. In a previous study, a proof of concept prototype of an electrochemical catheter hub (e‐catheter hub) was developed to verify electrochemical generation of HOCl within the catheter hub and In Vitro efficacy tested against four bacterial isolates derived from catheter‐related infection (Cano et al. 2021). The initial prototype e‐catheter hub utilized platinum (Pt) wires and was operated using a commercial potentiostat. However, commercial potentiostats are bulky and unsuitable for clinical or In Vivo use. Additionally, there is a need to evaluate and identify materials with properties supporting long‐term performance and feasibility of e‐catheters, including corrosion resistance, biocompatibility, and mechanical strength and flexibility.

Here, an e‐catheter hub was designed to deliver continuous In Situ HOCl using a battery‐powered micropotentiostat (MP). The e‐catheter hub was tested using three electrode materials—titanium (Ti), Pt, and gold (Au)—to assess HOCl generation and biocidal efficacy. Linear sweep voltammetry and microelectrodes were used to determine onset potentials for each electrode. Short‐ and long‐term HOCl generation capabilities of each material were also evaluated. Antimicrobial activity was tested against *Acinetobacter baumannii* ATCC‐17978, a multidrug‐resistant bacterial strain which represents a species associated with CLABSI. Additionally, performance of the MP‐powered e‐catheter hub was compared to that of a commercial potentiostat to determine the MP's suitability for clinical application.

## 2 Materials and Methods

### 2.1 Blank Catheter Hub Model

**Figure 1:**
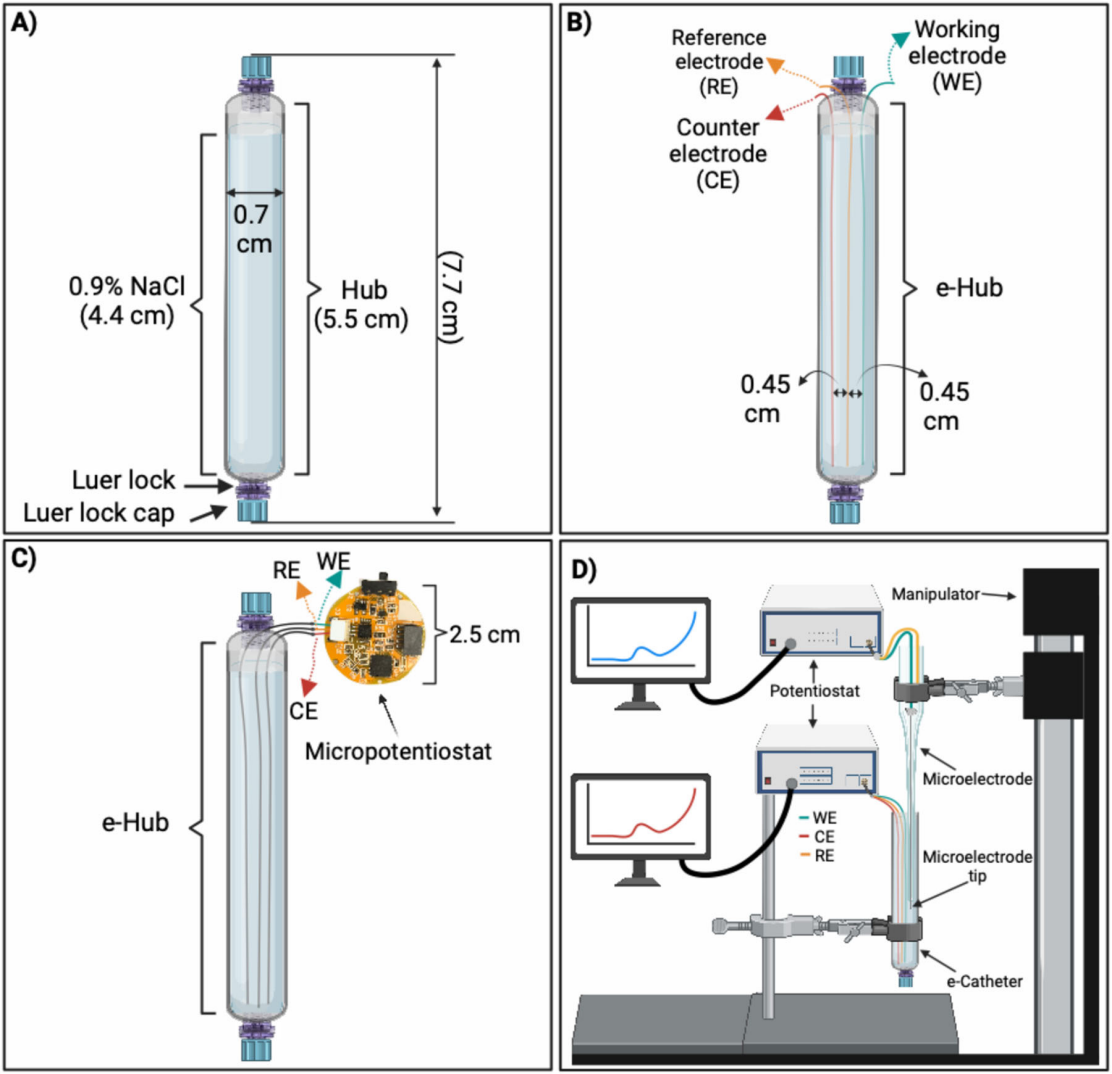
Schematic of a (A) blank catheter hub, (B) non‐polarized e‐catheter hub, (C) polarized e‐catheter hub operated by a micropotentiostat, and (D) HOCl microelectrode setup.

A blank catheter hub (wire‐free hub) was constructed using 5.5‐cm long disposable borosilicate glass Pasteur pipettes with an inner diameter of 0.54 cm (Fisher Scientific, Cat. No. 1367820D, Hanover Park, IL) (Figure 1A). Male Luer lock barb connectors (Qosina Corp., Ronkonkoma, NY) were inserted at both ends of the Pasteur pipettes using GE GE012A Silicone (GE Sealants, OH). Each end was capped with a polypropylene male Luer plug adapter (Qosina Corp., Ronkonkoma, NY).

### 2.2 Electrochemical Catheter Hub Model

The e‐catheter hub was constructed by inserting two 10‐cm (0.025‐cm diameter) wires made of Ti (Thermo Scientific Chemicals, Cat. NO. 010396.H2, MA), Pt (California Wire, Cat. NO. M457990, CA), or Au (California Wire, Cat. NO. M708250, CA), into the hub to serve as a working electrode (WE) and counter electrode (CE). Additionally, a 10‐cm (0.025‐cm diameter) silver (Ag) wire (Thermo Scientific Chemicals, Cat. NO. 041355.H4, MA), custom‐converted to a silver/silver chloride‐plated wire (Ag/AgCl), was inserted to function as quasi‐reference electrode (QRE). The QRE was calibrated against a standard Ag/AgCl reference electrode. The potential difference between the QRE and the standard Ag/AgCl electrode was ~0.085 V. This offset was accounted for in the experimental setup. As a result, when a potential of 1.5 V versus Ag/AgCl is reported, it corresponds to 1.415 V versus the QRE, accounting for a 0.085 V difference between the QRE and the standard Ag/AgCl electrode.

Wires were passed through the top end before the male Luer lock was attached and secured in place with a cap, leaving the electrodes exposed at one end (Figure 1B). Electrodes were arranged linearly along the length of the catheter, with the reference electrode (RE) positioned equidistant between the WE and the CE. The distance between the WE and RE, and the RE and CE, was 4.5 mm, with an accuracy of ± 0.2 mm based on carbon fiber composites digital caliper measurements. The bottom injection port (bottom cap) was used to introduce liquid before experimentation. Polarized e‐catheter hubs (Figure 1C) were operated by a small, lightweight MP developed by our group (Bozyel et al. 2025). Results were compared to those generated using a commercial Gamry^©^ Interface 1010T^TM^ potentiostat (Gamry^©^ Instruments, Warminster, PA).

Hubs were autoclaved at 121°C for 30 min, with the top and bottom ports left slightly open to prevent deformation by internal pressure buildup. Following sterilization, each hub was filled with 1 mL of sterile 0.9% NaCl using a 1‐mL syringe inserted through the bottom injection port. During the injection process, the top cap was loosened to allow air to escape, thereby preventing pressure buildup.

### 2.3 Wearable Micropotentiostat

The MP was designed with an integrated circuit incorporating two operational amplifiers (Texas Instruments, LPV542), each consuming 490 nA of quiescent current and delivering a maximum output current of 14 mA at a supply voltage of 3.3 V. Powered by a 3 V coin battery, the MP supports supply voltages from 2.0 to 5.5 V. A boost switch‐mode voltage regulator (Texas Instruments, TPS61023) maintained a stable 3.3 V supply with 0.9 μA quiescent current with 92% efficiency. The schematic and printed circuit board (PCB) layout were designed in KiCAD, with PCBs fabricated by OSH‐Park. Components, including ICs and connectors, were soldered onto the top layer, while the battery holder was placed on the bottom layer. Software development and validation were performed using STMCubeMX and STMCubeIDE, with pin configurations and clock settings defined via the STMCubeMX interface and embedded C code written in STMCubeIDE (Bozyel et al. 2025). MPs were connected to the STEVAL‐BCN002V1D programming board (STMicroelectronics, Geneva, Switzerland) mounted onto a STM32F401RE board with a STM32 St‐Link v2 interface (STMicroelectronics) to program treatment schemes. The STM32CubeIDE program was used to write and edit the code in C to set the polarization potential and duration of MP operation. Then, the STM32CubeProgrammer program was used to upload written code into the MP. After treatment, the STM32 ST‐LINK utility program was used to extract current data from the MP. These procedures were repeated for each MP.

### 2.4 Determination of the Onset Potential for HOCl Generation by e‐catheter Hubs

Electrochemical experiments along with HOCl concentration measurements were conducted to determine the potential range for HOCl generation in the e‐catheter hub under sterile conditions (i.e., absent bacteria). Custom‐built microelectrodes with tip diameters < 20 µm, operated via a commercial Gamry^©^ Interface 1010E^TM^ potentiostat (Gamry^©^ Instruments), were used to measure HOCl concentrations near the WE surface (Ti, Pt or Au) inside e‐catheter hub (Figure 1D). Construction and operation of microelectrodes have been described in previous publications (Atci et al. 2016; Istanbullu et al. 2012; Kiamco et al. 2019). A micromanipulator (MM33, Fine Science Tools) and stereoscope (M80, Leica) facilitated precise positioning of the microelectrode near the working electrode. Linear sweep voltammetry (LSV) was performed by gradually increasing WE potential from 0.0 V_Ag/AgCl_ to 2.0 V_Ag/AgCl_ at a scan rate of 10 mV/s. An independent commercial Gamry^©^ Interface 1010E^TM^ potentiostat and a G300 potentiostat (Gamry^©^ Instruments) were used to operate the HOCl microelectrode and the e‐catheter hub, respectively. The microelectrode tip was positioned ~23 mm deep inside the catheter, ~50 um from the WE. Since the microelectrode was parallel to the WE, measured concentrations near the electrode surface remained stable, enabling accurate evaluation of onset potential. However, based on previous modeling, concentration variations within the catheter are anticipated (Ozdemir et al. 2024). LSV data and corresponding HOCl concentrations near the electrode surface were analyzed to determine the onset potential for HOCl generation.

### 2.5 Measurement of Bulk pH and HOCl Concentrations

pH and HOCl measurements were performed after electrochemical HOCl generation via polarization of the e‐catheter hub (Figure 1C), which was filled with 1 mL of sterile 0.9% NaCl solution. Following HOCl generation, fluid in the e‐catheter hub (referred to as intraluminal fluid) was flushed into a sterile container. pH indicator strips (pH range: 4.0–7.0; ColorpHast, EMD Chemicals Inc., Burlington, MA) were applied to 20 μL aliquots of intraluminal fluid, and pH determined using a colorimetric scale provided by the manufacturer.

A free chlorine test (TNTplus 866; Hach, Loveland, CO) was used to measure HOCl concentrations in the intraluminal fluid. Following HOCl generation via polarization of e‐catheter hubs (Figure 1C), intraluminal fluid (0.980 mL) was mixed with 7 mL sterile water to fill test vials. Free chlorine concentrations were measured using a DR1900‐01H portable spectrophotometer (Hach, Loveland, CO). Measured free chlorine concentrations (mg/L) were adjusted to account for dilution. HOCl concentrations, expressed in millimolar (mM), were calculated based on the dissociation equilibrium of HOCl/OCl⁻ at the measured pH for each replicate (Black, & Corporation 2009). The free chlorine test had a detection limit of 0.05 mg/L, equivalent to 0.0056 mM HOCl at pH 5.5 and 25°C, corresponding to the average pH observed.

### 2.6 Activity of e‐catheter Hubs in Infection Prevention

To evaluate antimicrobial activity of e‐catheter hubs, a clinical isolate of *A. baumannii* ATCC‐17978, obtained from a catheter‐associated infection, was tested. This species was chosen due to its ability to persist on hospital surfaces and equipment for extended periods, its association with infections in critically ill patients, and its resistance to multiple antibiotics (Alsan and Klompas 2010; Ayoub Moubareck and Hammoudi Halat 2020). *A. baumannii* ATCC‐17978 was subcultured from frozen aliquots onto trypticase soy agar (TSA) and incubated at 37°C overnight. A single colony was transferred to 3 mL trypticase soy broth (TSB) and incubated at 37°C on an orbital shaker at 120 rpm for 4–5 h to achieve a 0.5 McFarland standard (~1.5 × 10^8^ colony forming units [CFU]/mL). The bacterial suspension was centrifuged, and the supernatant discarded. This pellet was resuspended in 1 mL of sterile 0.9% NaCl via vortexing. The homogenized suspension was diluted in sterile 0.9% NaCl to obtain a bacterial inoculum of ~2.5 × 10^4^ CFU/mL.

### 2.7 Experimental Setup

Each experimental replicate included one blank catheter hub (wire‐free hub) and two e‐catheter hubs (non‐polarized and polarized), each filled with 1 mL of 0.9% NaCl solution containing an inoculum of a ~2.5 × 10⁴ CFU/mL. The blank catheter hub (Figure 1A) served as a control to assess effects of the presence of Ti, Pt, Au, or Ag/AgCl wires in the hubs. Ti, Pt, and Au were chosen based on biocompatibility, electrochemical efficiency, corrosion resistance, electrical stability, and suitability for long‐term use, making them ideal for functional requirements of the e‐catheter hub. Ti has excellent corrosion resistance and forms a stable passive oxide layer during electrolysis, ensuring durability even under harsh conditions. Its biocompatibility also makes it suitable for applications where minimizing contamination is crucial (Radovanović et al. 2020). Pt was selected for biocompatibility, electrical conductivity, and durability, along with chemical inertness and surface integrity, attributes which make it suitable for biomedical applications (Sinitsyna et al. 2018). Moreover, Pt was initially chosen because previous work on e‐catheter hubs demonstrated excellent electrochemical performance and stability (Cano et al. 2021). Au is used for bioelectronic applications due to its electrical conductivity, chemical stability, and biocompatibility (Matarèse et al. 2018).

Among the e‐catheter hubs, the non‐polarized e‐catheter hub (Figure 1B) contained wires but had no applied potential, serving as a control for the polarized e‐catheter hub (Figure 1C). All hubs were placed in an incubator at 37°C. The polarized e‐catheter hub was connected to a commercial potentiostat or MP, with the WE (Ti, Pt or Au) polarized at 1.5 V_Ag/AgCl_. This potential was determined from LSV data obtained using microelectrodes. Polarization of the WE was performed over 1 to 24 h to determine the optimal HOCl generation time. Based on the results (data not shown), a 3‐h polarization period was selected as it demonstrated effective HOCl generation and antimicrobial activity for e‐catheter hubs with all electrodes. Following the 3‐h polarization period, hubs remained in the incubator for an additional 21 h, resulting in a total treatment time of 24 h.

### 2.8 Quantification of Intraluminal Bacterial Cells Following e‐catheter Hubs Treatment

After 24 h of treatment (i.e., 3 h of polarization followed by 21 h of non‐polarization), polarized e‐catheter hubs were disconnected from the commercial potentiostat or MPs. Each hub was aseptically uncapped, and its intraluminal fluid allowed to passively drain into a sterile 15‐mL Falcon tube under atmospheric pressure and gravity. Next, each hub was rinsed and flushed with 1 mL sterile 0.9% NaCl into the corresponding Falcon tube using a 1‐mL syringe. An additional 1 mL of sterile 0.9% NaCl was added to each hub, which was then aseptically capped on both ends and sonicated for 5 min in a Bransonic 1510 Ultrasonic Cleaner/Bath with Heat (Fisher Scientific, Hanover Park, IL) to remove any biofilm on the hub surface. Following sonication, intraluminal fluid was drained into the corresponding Falcon tube, and a final rinse with 1 mL of sterile 0.9% NaCl performed to bring the total intraluminal fluid volume to 4 mL. Intraluminal fluid was centrifuged at 3000 g for 10 min, and the supernatant removed. The pellet was resuspended in 1 mL sterile 0.9% NaCl and subjected to quantitative culture through serial dilution and plating onto TSA. Bacterial concentrations were calculated as log_10_ CFU/mL, with a lower quantification limit of 1 log_10_ CFU/mL. For samples below this limit, 1 mL of TSB was added to the remaining intraluminal fluid, and bacterial growth assessed by turbidity (detection limit, 1 CFU/mL). If no growth was observed in the broth culture after 48 h, values were reported as 0 ± 0 log₁₀ CFU/mL.

### 2.9 Statistical Analysis

A Mann‐Whitney test was used to assess intraluminal bacterial concentrations after 24 h of treatment in blank, non‐polarized, and polarized e‐catheter hubs (operated with either a commercial potentiostat or MPs, comparing results, which were analyzed as nonparametric data, to the initial inoculum. Each experiment was conducted in quadruplicate, with results presented as means with standard deviations (for quadruplicates). Statistical tests were two‐sided, with significance defined at *p* < 0.05. Analyses were conducted using GraphPad Prism 10 software.

## 3 Results and Discussion

### 3.1 Electrochemical Characterization

**Figure 2:**
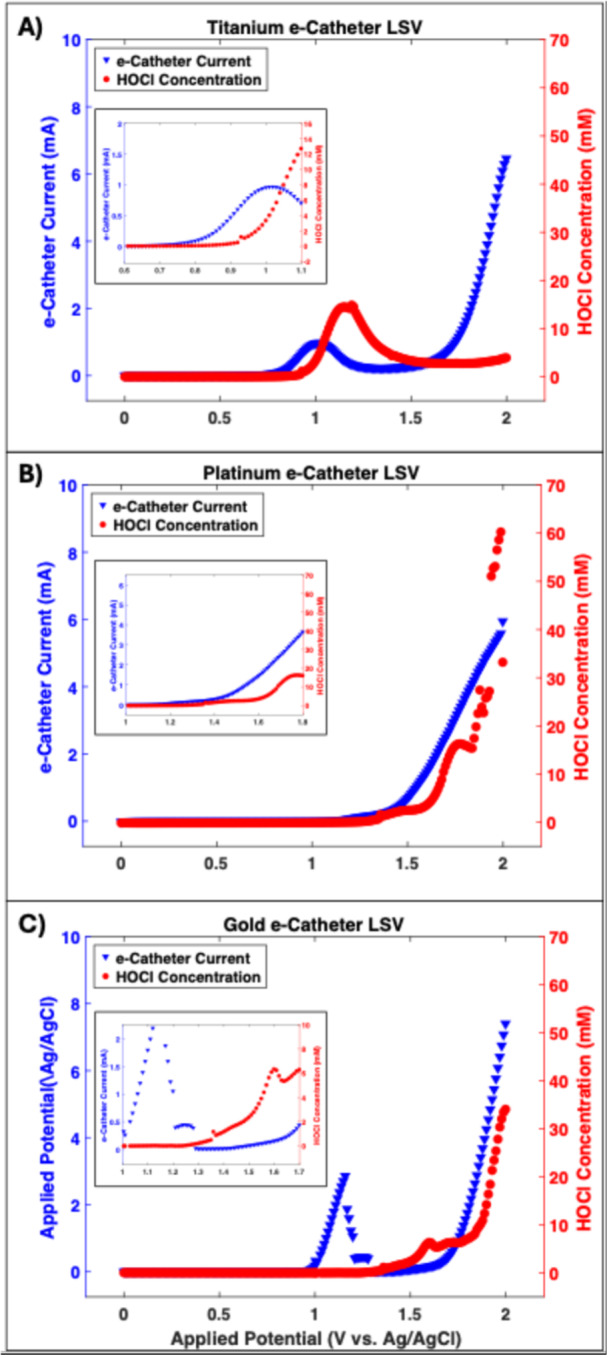
Linear sweep voltammetry and surface HOCl generation of e‐catheter hub, with WEs composed of (A) Ti, (B) Pt and (C) Au scanned at a rate of 10 mV/s.

Initial electrochemical studies were conducted using e‐catheter hubs with Ti, Pt, or Au to identify anodic regions that generate HOCl. These experiments, designed for consistency and performance evaluation of different electrode materials, utilized e‐catheter hubs filled with 1 mL of 0.9% NaCl without bacteria. Production of HOCl by e‐catheter hubs was assessed by monitoring its concentration during LSV using microelectrodes positioned near the surface of the WE (Figure 1D). Figure 2A–C shows HOCl concentration profiles for e‐catheter hubs with Ti, Pt, or Au electrodes as the applied potential varies from 0 to 2 V_Ag/AgCl_. For the e‐catheter hub with Ti electrodes (serving as WEs and CEs), HOCl generation initiated at ~0.75 V_Ag/AgCl_, with a peak concentration of 15 mM observed at 1.2 V_Ag/AgCl_ (Figure 2A). When Pt electrodes were used, the onset potential increased to ~1.0 V_Ag/AgCl_, reaching a maximum HOCl concentration of 61 mM at 2 V_Ag/AgCl_ (Figure 2B). Similarly, the Au‐electrode configuration demonstrated an onset potential of ~1.3 V_Ag/AgCl_, with a peak HOCl concentration of 34 mM at 2 V_Ag/AgCl_ (Figure 2C). These results demonstrate distinct onset potentials for HOCl generation with different electrode materials in the e‐catheter hub.

Notably, as the applied formal potential exceeded 1.45 V_Ag/AgCl_ (pH = 5.5), data indicated that chloride ions were oxidized directly to HOCl without the intermediate formation of Cl₂ gas, as represented by Equation 4 (Murrieta and Nava 2021):

(4) $$2{\text{Cl}}^{-}+H_{2}O⇄\text{HOCl}+H^{+}+{\text{Cl}}^{-}+2{\text{e}}^{-}\;E^{o^{\prime }}=1.45V_{Ag/AgCl}$$

Based on the data presented in Figure 2, a potential of 1.5 V_Ag/AgCl_ was selected as optimal for sustained HOCl production during subsequent experiments. This aligns with prior findings (Cano et al. 2021), which established 1.5 V_Ag/AgCl_ as effective for consistent HOCl generation. A potential higher than onset potentials found in Figure 2 was selected, allowing consistent generation of HOCl, ensuring efficient chloride ion conversion to HOCl without intermediate Cl₂ formation. This concept is further supported by the observation that none of the experimental pH values fell within the acidic range (pH < 4), where Cl₂ typically forms (Wang et al. 2007). Instead, all measured pH values were above 5 (5.2–5.8), suggesting the absence of Cl₂ and reinforcing direct conversion of chloride ions to HOCl under the experimental conditions studied. While HOCl is the primary antimicrobial agent generated, other reactive species, such as protons (H⁺), hypochlorite (OCl⁻), chlorite (ClO₂⁻), and chlorate (ClO₃⁻), may form as byproducts during electrolysis (Ozdemir et al. 2024). However, given the system's pH range, HOCl is the dominant species due to its stability in slightly acidic conditions. Its concentration is highest between pH 4 and 6, whereas at higher pH (8.5–10), OCl⁻ becomes more prevalent (da Cruz Nizer et al. 2020). Although byproducts exist, bacterial killing is likely secondary to HOCl, with efficacy primarily driven by its concentration and stability.

**Figure 3:**
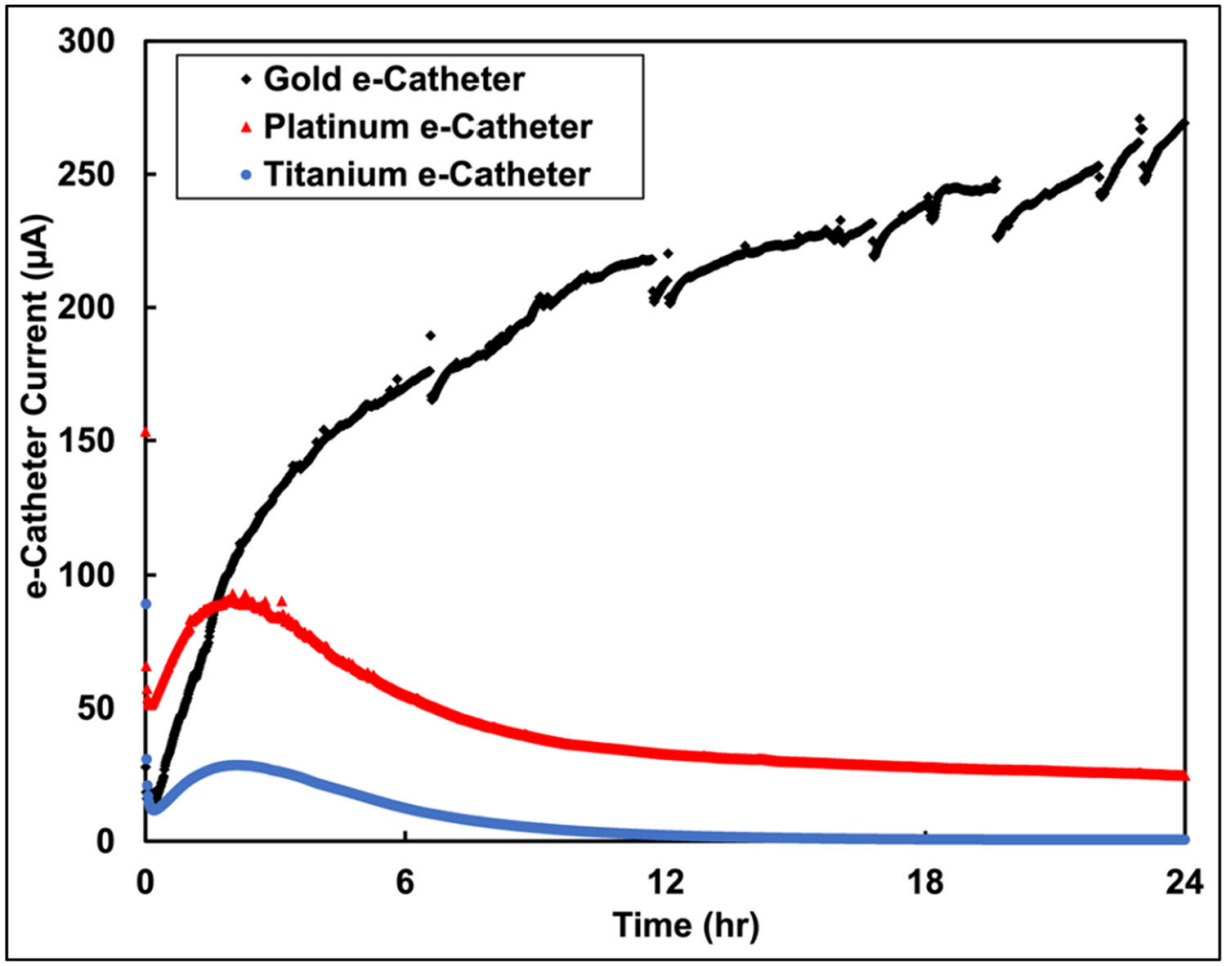
Chronoamperometry scan of e‐catheter hubs with Au, Pt, and Ti electrodes with WEs polarized at 1.5 V_Ag/AgCl_ over 24 h for HOCl generation.

Twenty ‐four‐h chronoamperometry (CA) experiments were run to assess amount of HOCl generated from e‐catheter hubs with Au, Pt, or Ti electrodes. As shown in Figure 3, chronoamperometric data revealed variations in current responses among electrode materials, demonstrating differences in efficiency for HOCl generation. e‐catheter hubs with Au electrodes exhibited a steady increase in current over time, reaching ~270 µA after 24 h and resulting in ~5 mM of HOCl. This trend suggests a robust and sustained oxidation reaction, indicative of efficient HOCl production. In contrast, e‐catheter hubs with Pt electrodes demonstrated an initial rapid current increase, peaking at ~92 µA within the first 3 h, followed by a gradual decline. This pattern suggests an initial burst of HOCl production that stabilized at lower rates, ultimately yielding ~0.3 mM of HOCl. Meanwhile, e‐catheter hubs with Ti electrodes exhibited lower current responses, remaining below 28 µA throughout the experiment and yielding no detectable HOCl (0 mM). The limited current response of Ti electrodes reflects their lower electrochemical activity under the applied conditions.

Based on the CA scans (Figure 3), a 3‐h polarization period was selected for further experimentation, as preliminary data indicated that this duration yields the maximum concentration of HOCl produced from Ti or Pt electrodes. Given that Ti has excellent corrosion resistance and is used in medical devices (Hanawa 2019), optimizing performance of the Ti electrode for production of HOCl was logical. The resistance of Ti to corrosion is attributed to its ability to form a stable oxide layer (TiO₂) when exposed to an oxidizing environment, even at low potentials (Dubent and Mazard 2019). During polarization, Ti undergoes oxidation, leading to rapid formation of a surface TiO₂ passivation layer. This passivation layer serves as a protective barrier, preventing corrosion and limiting transfer of electrons across the electrode interface (Wang et al. 2016). While the passivation layer maintains the electrode's structural integrity, it impedes electrochemical reactions by limiting electron flow, which is needed for sustained HOCl generation. As the passivation layer thickens, it increasingly restricts electron transfer across the electrode interface, leading to a decrease in HOCl production over extended polarization periods. For this reason, a 3‐h polarization period was selected to achieve optimal current and HOCl production before significant passivation occurred. Prolonging polarization to 24 h resulted in current limitations due to buildup of the TiO₂ layer, which further restricted HOCl generation. These findings demonstrate the importance of balancing the protective benefits of the TiO₂ layer with its impact on efficiency of electrochemical reactions. Therefore, in 3 h of polarization, production of HOCl was observed to be substantial across all electrodes, confirming that this duration effectively optimizes electrochemical activity before the thickening passivation layer on the Ti electrode begins to hinder the efficiency of HOCl generation.

### 3.2 Intermittent HOCl Generation

Initially, e‐catheter hubs were injected with 1 mL of 0.9% NaCl and polarized at 1.5 V_Ag/AgCl_ for 3 h by a commercial potentiostat, after which HOCl concentrations were measured. This was followed by a 21‐h rest period. After each rest period, 1 mL of fresh saline solution was added, and e‐catheter hubs polarized again for 3 h, followed by subsequent HOCl measurement. This cycle was repeated daily for 5 days to evaluate the ability of e‐catheter hubs to maintain sustained HOCl concentrations. Due to the inefficiency of Ti electrodes during prolonged polarization, this alternative intermittent HOCl generation strategy was developed for extended use over 5 days.

Bulk pH and HOCl concentrations were measured after electrochemical HOCl generation rather than being continuously monitored. pH variations in the system are influenced by several factors, including diffusion and H⁺ consumption; accordingly, pH changes and HOCl production can be decoupled. During HOCl generation, H⁺ ions are produced as a byproduct of the reaction shown in Equation 4. H⁺ ions do not accumulate uniformly due to diffusion, which causes them to move from regions of high concentration near the electrode surface to areas of lower concentration in the bulk solution. Additionally, H⁺ ions can be consumed in subsequent reactions, such as recombination with OH⁻ to form water or buffering effects if buffering species are present in the solution. In previous work (Kiamco et al. 2019), it was observed that HOCl generation on electrodes did not result in significant pH changes. Furthermore, in a recent modeling study of the e‐catheter system (Ozdemir et al. 2024), it was demonstrated that pH remains nearly constant throughout the device, likely due to the rapid diffusion and consumption of H⁺ ions.

**Figure 4:**
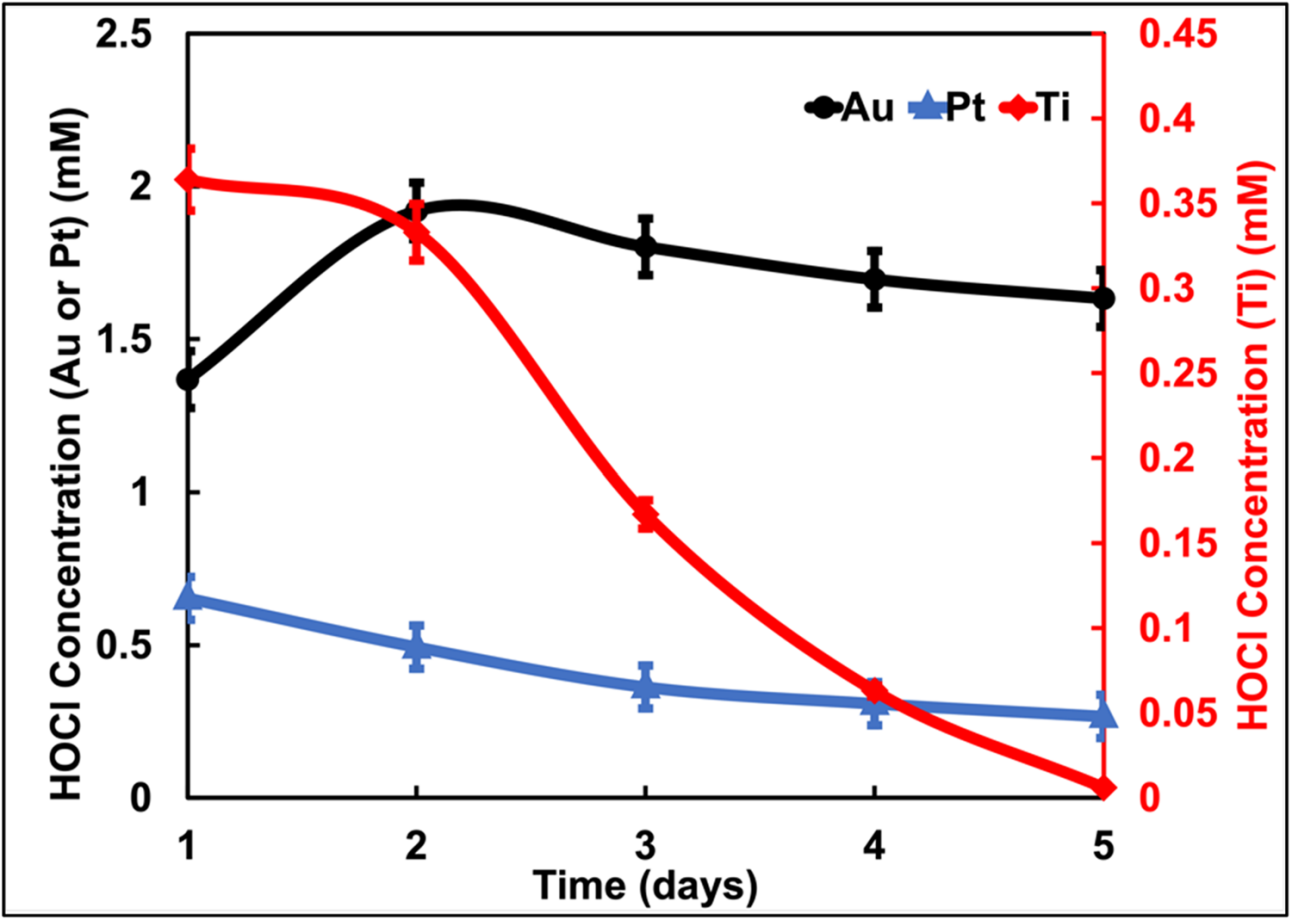
HOCl generation within e‐catheter hubs intermittently over 5 days using a commercial potentiostat with Ti, Pt, and Au electrodes.

Figure 4 reveals that while Ti electrodes can be used intermittently for HOCl production, their performance declines over time, likely due to formation of a passivation layer. To address this limitation and improve HOCl generation in the hub, Pt and Au electrodes were tested. Pt electrodes have been previously shown to generate HOCl efficiently and exhibit strong biocidal activity when integrated into e‐catheter hubs (Cano et al. 2021). As shown in Figure 3, Au electrodes have not been tested before but are known for their electrochemical stability.

Figure 4 demonstrates that both Pt‐ and Au‐equipped e‐catheter hubs generated higher HOCl concentrations compared to Ti. Notably, Au electrodes produced HOCl concentrations greater than those produced from Pt or Ti electrodes. These findings establish that for applications requiring e‐catheter hubs to operate continuously over extended periods (> 5 days), hubs with Au electrodes offer the most favorable option due to superior and sustained HOCl generation.

Additionally, Figure 4 shows that maximum concentrations achieved with the Au electrode remained below the solubility limit of chlorine gas (Cl_2_; 60–82 mol/m³) (Awakura et al. 1990). This indicates that Cl₂ is not produced during the process, as described in Equation 1. Instead, chloride ions (Cl⁻) are directly oxidized to generate HOCl, as represented in Equation 4 (Murrieta and Nava 2021), which aligns with the LSV data in FIGURE 2, which showed no evidence of Cl₂ formation, further confirming the selective oxidation of Cl⁻ to HOCl.

### 3.3 Comparison of HOCl Generation in e‐catheter Hubs Operated by a Commercial Potentiostat and a Micropotentiostat

A focus of this study was to evaluate the capability and efficacy of a previously developed MP in operating an e‐catheter hub, with a focus on HOCl generation and biocidal efficacy. To achieve this, e‐catheter hubs with Ti, Pt, or Au electrodes were operated for 3 h at 1.5 V_Ag/AgCl_ by a commercial potentiostat to generate HOCl. In a subsequent set of experiments, the commercial potentiostat was replaced with MPs. This comparative approach aimed to validate the MP's reliability and assess its potential for future applications.

Table 1 shows that the e‐catheter hubs operated by a commercial potentiostat or a MP generated similar HOCl concentrations across Ti, Pt, or Au electrode materials, with no statistically significant differences (*p* > 0.05). Overall, these findings demonstrate that the MP effectively operates the e‐catheter hub, generating HOCl concentrations comparable to those achieved with a commercial potentiostat. This makes the MP‐operated e‐catheter hub a suitable candidate for In Vivo application, where portability, consistent HOCl generation, and infection prevention are critical requirements., where portability, consistent HOCl generation, and infection prevention are critical requirements.

**Table 1:** Mean and standard deviation of electrochemically generated HOCl by e‐catheter hubs equipped with Ti, Pt, or Au electrodes operated at 1.5 V_Ag/AgCl_ for 3 h by a commercial potentiostat and a micropotentiostat (MP) (*n* = 3).

Electrode	[HOCl] (mM) Operated by a commercial potentiostat	[HOCl] (mM) Operated by a micropotentiostat
Ti	0.45 ± 0.06	0.48 ± 0.03
Pt	0.78 ± 0.07	0.73 ± 0.03
Au	2.35 ± 0.24	2.46 ± 0.34

HOCl, while effective against pathogens, can potentially cause cytotoxic effects in mammalian cells through oxidative modifications and enzyme disruption, leading to apoptosis or necrosis (Singer et al. 2024). The described system mitigates these risks by controlling concentration, localizing delivery, and using biocompatible materials. Further, in a previous study (Ozdemir et al. 2024), methods were described to modulate HOCl concentrations by adjusting WE length, exposure time, and applied potential.

### 3.4 Comparison of Biocidal Efficacy of e‐catheter Hubs Operated by a Commercial Potentiostat and a Micropotentiostat

**Figure 5:**
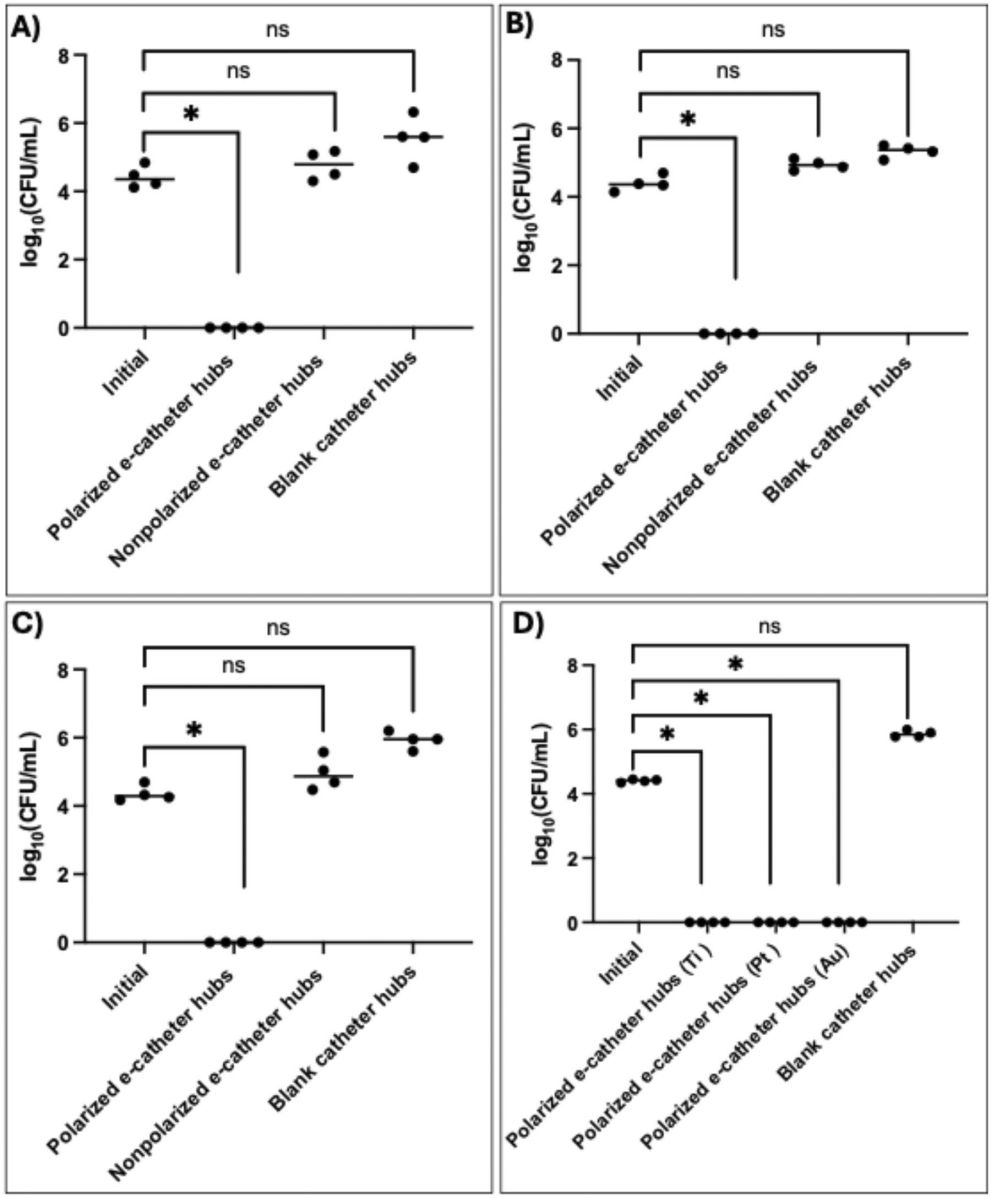
Biocidal efficacy of HOCl generated electrochemically by e‐catheter hubs (*n* = 4) against *A. baumannii* ATCC‐17978, operated using a commercial potentiostat and equipped with (A) Ti, (B) Pt, and (C) Au electrodes. (D) Polarized e‐catheter hubs utilizing Ti, Pt, or Au wires operated by MPs.

After confirming that the developed e‐catheter hub generated HOCl, its activity was evaluated against *A. baumannii* ATCC‐17978. Polarized e‐catheter hubs, configured with WEs and CEs made of Ti, Pt or Au and a reference electrode made of Ag/AgCl, were operated using a commercial potentiostat. These configurations reduced the bacterial load below the limit of detection (0 ± 0 log_10_), achieving a mean reduction of 4.40 ± 0.03 log_10_ CFU/mL (*p* < 0.05) compared to the initial inoculum, as illustrated in Figure 5A–C. This reduction emphasizes the device's ability to mitigate infection within the e‐catheter hubs after just 3 h of polarization. In contrast, control groups (blank catheter hubs and non‐polarized e‐catheter hubs) exhibited no reduction in cell viability after 24 h, confirming that the bacterial load reduction was only due to the polarized e‐catheter hub. Subsequently, MPs were used to operate e‐catheter hubs, and biocidal activity was tested using the same protocol as for hubs operated with a commercial potentiostat (Figure 5D). Polarized e‐catheter hubs incorporating Ti, Pt, and Au electrodes were used, with blank catheter hubs serving as controls. Non‐polarized e‐catheter hubs were excluded from this comparison, as their results are shown in Figure 5A–C. All e‐catheter hubs with Ti, Pt, and Au electrodes operated using MPs resulted in bacterial load reductions below the limit of detection (0 ± 0 log_10_), achieving a mean reduction of 4.40 ± 0.05 log_10_ CFU/mL (*p* < 0.05) compared to the initial inoculum. These findings demonstrate that e‐catheter hubs operated with MPs exhibit biocidal activity comparable to those operated by a commercial potentiostat. Use of the MPs achieved similar reductions in bacterial viability across e‐catheter hubs with Ti, Pt, and Au electrodes, further showcasing the MP's versatility in producing HOCl under varied conditions. The consistent performance across both potentiostat‐types demonstrates the potential of MPs as a wearable alternative, suited for In Vivo applications where compact and portable devices are essential.

## 4 Conclusion

This study successfully developed a nonantibiotic, animal‐ready e‐catheter hub operated by a lightweight, battery‐powered, wearable MP to address the challenge of intraluminal infections in CVCs, including those caused by multidrug‐resistant organisms. e‐catheter hubs generated tunable HOCl concentrations, achieving significant antimicrobial activity, with a mean reduction of 4.40 ± 0.05 log_10_ CFU/mL in *A. baumannii* ATCC‐17978 (*p* < 0.05). Among tested electrode materials, Au electrodes delivered sustained HOCl concentrations, maintaining high performance for up to 5 days, making them ideal for long‐term applications. Pt electrodes exhibited comparable HOCl generation to Au, further demonstrating their suitability for extended use. While Ti electrodes were less e effective for sustained HOCl generation due to anodization, they achieved comparable biocidal activity during a 3‐h polarization period, positioning them as viable for short‐term or intermittent applications. Integration of the wearable, battery‐powered MP ensures continuous and reliable operation of e‐catheter hubs, making the system practical for clinical settings. The MP demonstrated biocidal activity like that of a commercial potentiostat, supporting its potential for scalable, In Vivo applications. The described e‐catheter hub provides a potential step forward in infection prevention, offering a localized, nonantibiotic solution to potentially mitigate the risk of CLABSIs. This addresses the need for innovative solutions to combat infections without relying on antibiotics, addressing the fight against antibiotic resistance in healthcare settings.

## Author Contributions

Conceptualization: Haluk Beyenal and Robin Patel. Methodology: Majid Al‐Qurahi, Derek Fleming, Won‐Jun Kim, Ibrahim Bozyel. Writing‐original draft preparation: Majid Al‐Qurahi. Writing‐review and editing: all authors. Project administration: Haluk Beyenal and Robin Patel. Funding acquisition: Haluk Beyenal and Robin Patel.

## Conflicts of Interest

The authors declare no conflicts of interest.

## Data Availability

The data that support the findings of this study are available on request from the corresponding author. The data are not publicly available due to privacy or ethical restrictions.
